# The transcriptomic and evolutionary signature of social interactions regulating honey bee caste development

**DOI:** 10.1002/ece3.1720

**Published:** 2015-10-08

**Authors:** Svjetlana Vojvodic, Brian R. Johnson, Brock A. Harpur, Clement F. Kent, Amro Zayed, Kirk E. Anderson, Timothy A. Linksvayer

**Affiliations:** ^1^ Center for Insect ScienceUniversity of ArizonaTucsonArizona; ^2^ Department of Biological SciencesRowan UniversityGlassboroNew Jersey; ^3^ Department of EntomologyUniversity of CaliforniaDavisCalifornia; ^4^ Department of BiologyYork UniversityTorontoOntarioCanada; ^5^ Carl Hayden Bee Research CenterUSDATucsonArizona; ^6^ Department of EntomologyUniversity of ArizonaTucsonArizona; ^7^ Department of BiologyUniversity of PennsylvaniaPhiladelphiaPennsylvania; ^8^ Janelia Research CampusHHMIAshburnVAUSA

**Keywords:** Extended phenotype, indirect genetic effects, interacting phenotypes, social evolution

## Abstract

The caste fate of developing female honey bee larvae is strictly socially regulated by adult nurse workers. As a result of this social regulation, nurse‐expressed genes as well as larval‐expressed genes may affect caste expression and evolution. We used a novel transcriptomic approach to identify genes with putative direct and indirect effects on honey bee caste development, and we subsequently studied the relative rates of molecular evolution at these caste‐associated genes. We experimentally induced the production of new queens by removing the current colony queen, and we used RNA sequencing to study the gene expression profiles of both developing larvae and their caregiving nurses before and after queen removal. By comparing the gene expression profiles of queen‐destined versus worker‐destined larvae as well as nurses observed feeding these two types of larvae, we identified larval and nurse genes associated with caste development. Of 950 differentially expressed genes associated with caste, 82% were expressed in larvae with putative direct effects on larval caste, and 18% were expressed in nurses with putative indirect effects on caste. Estimated selection coefficients suggest that both nurse and larval genes putatively associated with caste are rapidly evolving, especially those genes associated with worker development. Altogether, our results suggest that indirect effect genes play important roles in both the expression and evolution of socially influenced traits such as caste.

## Introduction

The social insect sterile worker caste is the archetypal example of reproductive altruism that initially puzzled Darwin (1859) and spurred Hamilton (1964) to develop kin selection theory. Kin selection theory presupposes the existence of genes that are expressed in one individual but have fitness effects on relatives (Hamilton 1964). Despite this clear focus of social evolution theory on socially acting genes, empirical studies of the genetic basis of social insect traits, including caste, have widely overlooked the contribution of such genes with indirect effects that are expressed in one individual but affect the traits of social partners (Moore et al. 1997; Linksvayer 2015).

Honey bee female caste is considered to be an exemplar polyphenism, whereby the expression of alternate queen and worker morphs is controlled by environmental cues (Evans and Wheeler 1999). Unlike some other well‐studied polyphenisms that are controlled by simple abiotic factors such as temperature or photoperiod (Nijhout 2003), honey bee queen–worker dimorphism critically depends on social control of larval development by adult nestmates (Linksvayer et al. 2011). *In vitro* rearing studies demonstrate that in the absence of social control, queen–worker dimorphism disappears and a continuous range of phenotypes are produced (Linksvayer et al. 2011).

**Figure 1:**
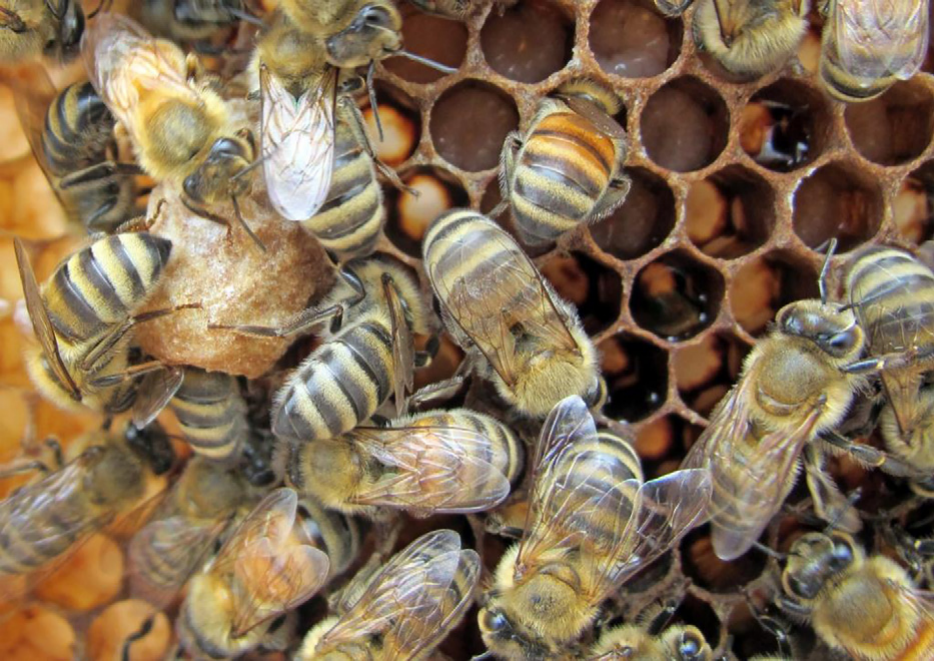
Honey bee workers rear most of their larvae in hexagonal cells (upper right) provisioned with a relatively small quantity of food so that the larvae develop into new workers. A few larvae are reared as new queens in larger queen cells (center left) that are newly constructed and provisioned with more and qualitatively different brood food.

Honey bee colonies only rear new queens during specific life‐history stages, for example, in the spring when the colony is large enough to split in half, or upon the death of the current queen. Queen rearing is an emergent, colony‐level process involving the coordinated activities of hundreds or thousands of adult workers. Necessary steps include the construction of special queen cells by nurse bees (Fig. 1), distinct provisioning behavior of nurses coupled with distinct qualitative and quantitative differences in the nutrition fed to queen‐ and worker‐destined larvae (colloquially known as “royal jelly” vs. “worker jelly”) (Haydak 1970; Brouwers et al. 1987), the larval developmental response to these environmental signals, and finally, selection by nurses of a subset of larvae in queen cells to be reared to adulthood (Hatch et al. 1999).

Previous studies of the genetic basis of caste and other social insect traits have mainly used a conventional genetic approach, which seeks direct links between an individual's genotype or patterns of gene expression and its phenotype (Evans and Wheeler 1999; Barchuk et al. 2007; Chandrasekaran et al. 2011). These studies have led to exciting progress in our understanding of the endogenous molecular genetic, epigenetic, and endocrine basis of alternate larval developmental trajectories in response to socially controlled nutritional inputs (Evans and Wheeler 1999; Barchuk et al. 2007; Kucharski et al. 2008; Foret et al. 2012). For example, experimental gene knockdown studies demonstrate that insulin/TOR pathways mediating physiological and developmental responses to the nutritional environment strongly affect an individual's caste fate (Patel et al. 2007; Mutti et al. 2011; Wolschin et al. 2011). However, the conventional approach has limited ability to identify exogenous socially acting genes (Hahn and Schanz 1996; Wolf and Moore 2010).

As a result, the contribution of genes expressed in adult nestmates (e.g., nurses and foragers) to the genetic basis and evolution of the honey bee caste developmental program has received relatively little attention. Two exogenous, nurse‐produced royal jelly proteins have been implicated as promoting queen development (Kamakura 2011; Huang et al. 2012). These and other protein‐coding genes are very highly expressed in nurse hypopharyngeal and mandibular glands (Santos et al. 2005; Jasper et al. 2014), and different proportions of these glandular secretions are combined with sugars and proteins and fed to larvae, depending on the age and caste trajectory of the larva (Haydak 1970; Brouwers et al. 1987). Social control of caste development means that exogenous molecular factors expressed in adult nestmates may make up a significant portion of the colony‐level gene regulatory network underlying queen development (Linksvayer et al. 2011). Indeed, quantitative genetic studies have demonstrated that the expression of honey bee caste and caste‐related traits depends on both larval genotype and nurse genotype (Osborne and Oldroyd 1999; Beekman et al. 2000; Linksvayer et al. 2009a,b).

The interacting phenotype framework was developed to study the quantitative or statistical effects of social interactions on trait variation (Moore et al. 1997; Bleakley et al. 2010; McGlothlin et al. 2010; Wolf and Moore 2010). Under this conceptual framework, an individual's traits depend directly on its own genes (direct genetic effects) and indirectly on its social partners' genes (indirect genetic effects) (Moore et al. 1997). In this study, we extend the interacting phenotype approach and examine transcriptomic differences associated directly with developing larvae and indirectly with the effects of nurses in their social environment. Thus, instead of searching only for associations between a developing larva's own patterns of gene expression and its caste fate, we also search for associations between larval caste fate and the gene expression profiles of caregiving nurses, with a goal of beginning to characterize the full colony‐level set of molecular interactions regulating reproductive caste (Linksvayer et al. 2012). Specifically, we used RNA sequencing of queen‐ and worker‐destined larvae as well as nurses collected in the act of feeding queen‐ and worker‐destined larvae, respectively. We also determined whether there was evidence for behavioral and physiological specialization of nurses to feed queen‐ versus worker‐destined larvae, as such specialization is expected to strengthen the transcriptional signature of social effects on caste development.

Finally, we used a new honey bee population genomic dataset (Harpur et al. 2014) to compare rates of molecular evolution at the genes we identified as putatively being directly or indirectly associated with larval caste fate. All else equal, genes with indirect fitness effects (i.e., genes shaped by kin selection) are expected to evolve more rapidly than genes with direct fitness effects, as a result of relaxed purifying selection (Linksvayer and Wade 2009). Broadly, we predicted that nurse genes indirectly associated with caste, which are expected to be shaped by indirect selection, would have higher rates of molecular evolution than larval genes directly associated with caste, which are expected to be shaped by direct selection. Furthermore, we predicted that if both nurse and larval caste‐associated genes were subdivided into genes associated with worker development versus genes associated with queen development, the set of genes associated with worker development would be more rapidly evolving than the set of genes associated with queen development. Because honey bee workers are facultatively sterile, all genes associated with worker development should ultimately be shaped mainly by indirect selection. Altogether, the results of our study suggest that nurse‐expressed genes with indirect effects on larval caste fate play important roles in both the expression and evolution of honey bee caste.

## Methods

### Overview

In April 2011, we performed a preliminary study to determine whether individually marked workers were behaviorally or physiologically specialized on rearing new queens and workers. The main study, conducted in June 2011, was focused on collecting nurse and larval samples for RNA sequencing. Both studies were conducted at the USDA Carl Hayden Bee Research Center in Tucson, AZ. We used commercial *Apis mellifera* stock colonies to create 4‐frame observation hives. We constructed observation hives with a hinged plexiglass door over each frame on each side so that it was possible to gently open the door and collect nurse and larval samples without disturbing the colony. The studies mimicked emergency queen rearing that occurs in the days immediately following queen loss.

### Preliminary study of nurse behavioral specialization during queen and worker rearing

Our preliminary behavioral study used two replicate observation hives. Every 3 days beginning 24 days before the start of the study, we individually marked 400 newly emerged adult workers with a unique combination of numbered tag glued onto the mesosoma and an age‐specific abdomen paint mark, and we added 200 individually marked workers to each observation hive. Frames of known‐aged brood were produced by caging queens on empty frames for 24 h and then checking for the presence of eggs. Four days later, one frame with only similarly aged 1st instar larvae was placed into each observation hive, and the queen was removed to initiate emergency queen rearing. These frames were the source of young focal larvae, a fraction of which were reared as new queens, and the rest as workers. Within the first 2 days of queen removal, nurse workers build wax queen cells over young focal brood and begin provisioning these queen‐destined larvae differentially than worker‐destined larvae in worker cells (Fig. 1). We continually observed areas of the frame with focal brood that contained both queen cells and worker cells and recorded the date, time, and identity of nurses observed provisioning queen or worker cells (i.e., “royal nurses” or “worker nurses”). Feeding behavior was defined when workers had their head positioned deep enough into the worker or queen cell to be in contact with the larva and remained motionless except for a rhythmic motion of the abdomen for at least 5 sec.

### Transcriptomic profiling to identify larval and nurse genes associated with caste development

The main RNA sequencing study used three replicate observation hives. The setup followed the preliminary study, except that we collected samples of focal brood under both queen present and queen removed conditions. First, on the fourth day after introducing focal brood, samples of five 4th instar worker‐destined larvae and 20 nurses observed feeding 4th instar worker‐destined larvae were collected. Two days later, a new frame of same‐aged 1st instar larvae was added to each of the three observation hives, and each colony queen was removed in order to initiate emergency queen rearing. On the fourth day after introducing focal brood and removing the queen, we collected five 4th instar worker‐destined larvae from the frame of focal brood, and we collected 20 worker nurses in the act of feeding these 4th instar worker focal brood. Similarly, we collected 20 royal nurses in the act of provisioning 4‐day‐old queen cells. Finally, we collected five 4th instar queen larvae from the 4‐day‐old queen cells. After removal from the hive, samples were immediately frozen in liquid nitrogen and stored on dry ice. We chose to collect larval and nurse samples when the larvae were 4th instar because this is a period of very rapid larval growth (Haydak 1970; Evans and Wheeler 1999; Barchuk et al. 2007) as well as when differences in nurse provisioning are marked (Haydak 1970), even though most caste‐related characters are considered to be already determined by this stage (Dedej et al. 1998).

In total, we collected (1) worker larvae from colonies with a queen, (2) worker larvae from queenless colonies, (3) queen larvae from queenless colonies, (4) worker nurses from colonies with a queen, (5) worker nurses from queenless colonies, and (6) royal nurses from queenless colonies. Thus, for both larvae and nurses, there were three total conditions: two conditions associated with worker production (colonies with and without a queen) and one condition associated with queen production (colonies without a queen). Using these larval and nurse samples, we extracted RNA from four tissue types. First, we used whole larvae (L). For nurse samples, we dissected two head glands, hypopharyngeal glands (HPG) and mandibular glands (MG), and finally, we used the remaining head tissue (H, made up mostly of brain tissue, but also including salivary gland tissue). We separated these nurse tissues because the HPG and MG are the two main glandular sources of the proteinaceous brood food (e.g., royal jelly proteins) that regulates larval development (Haydak 1970; Brouwers et al. 1987; Schonleben et al. 2007). We reasoned that genes expressed in these glands and the brain could be associated with queen versus worker rearing.

### Nurse tissue dissections and mRNA sequencing

Nurse heads were thawed in RNA*later* (Qiagen), immediately dissected, and the three tissues (HPG, MG, H) collected and stored in RNA*later* at −80. HPG size is associated with gland activity, and HPG size (i.e., as measured by the diameter of HPG acini, which make up the HPG) changes as the nurse ages (Ohashi et al. 2000). To quantify HPG size variation between nurse samples, we took an image at 50× of a small subsample of each HPG, and subsequently, three haphazardly chosen HPG acini were measured at their widest point by an observer blind to the sample treatment.

RNA was extracted from individual larval samples and from tissue pooled from 5 nurses, for each of the three nurse tissue types, using Qiagen RNeasy kits. RNA concentration was quantified with Nanodrop, and final pools created by combining RNA from 5 larvae from each of the three replicate colonies (15 total larvae), or from a tissue from 20 nurses from each of the three replicate colonies (60 total nurses). Separate pools were created for each of the three conditions (worker‐associated in colonies with a queen; worker‐associated in colonies without a queen; queen‐associated in colonies without a queen) and four tissues (L, HPG, MG, H), resulting in 12 total pools.

Note that although we started with three replicate colonies, we pooled samples across these replicates to produce the 12 pools because sequencing 12 types of samples for each of 3 replicates was cost prohibitive. As a result, we ended up with limited to no replication (i.e., two replicates for each worker‐associated sample condition and no replicates for each queen‐associated sample condition). Even though the field has rapidly moved toward increasing replication (e.g., 2–3 or more replicates) as costs have dropped, most current RNA seq software packages are capable of making statistical inference with minimal or no biological replication given certain assumptions. For example, the mean–variance relationship for expression can be inferred across all genes instead of relying on a good estimate of variance in expression for each individual gene, based on multiple replicates (Anders and Huber 2010; Leng et al. 2013; Love et al. 2014). Such inference is expected not only to have decreased power but also to be affected by any random technical errors that may occur during the sequencing and analysis process. To minimize the impact of such errors, we focused our attention only on the most highly expressed genes that were observed to be similarly highly expressed in another recent RNA seq studies that studied replicate worker HPG and M tissue samples (Jasper et al. 2014). Furthermore, we mainly focus our attention on discussing overall patterns instead of focusing on individual genes.

RNA sequencing libraries were constructed at the University of Arizona Genetics Core, using RNA TruSeq library construction kits and Bioanalyzer RNAchips to check the library quality prior to sequencing. RNA samples were multiplexed on an Illumina HiSeq2000 with 6 samples per lane on two lanes with 100‐bp paired‐end reads. Sequences were postprocessed through trimmomatic to remove Illumina adapter sequences. Fastx and cutadapt software packages were used to remove reads with average quality scores <25, and the ends of reads were clipped so that the mean quality of the last five bases was >25. To control for initial variation in raw read number among samples within tissues, we used a standardized number of raw reads across all samples within each tissue.

### Differential gene expression analysis

We aligned the reads to the *Apis mellifera* genome build 4.5 (Elsik et al. 2014) using Tophat v2.04 (Trapnell et al. 2012) with Bowtie2 and default parameters. We used htseq‐count in the HTSeq (Anders and Huber 2010) Python Package with default parameters to obtain counts of read pairs mapped to the *A. mellifera* Official Gene Set 3.2 (Elsik et al. 2014). We subsequently used two different R v3.1.0 (www.r-project.org) packages to analyze differential gene expression, EBSeq v1.5.4 (Leng et al. 2013) and DEseq2 v1.4.5 (Love et al. 2014). DEseq2 identifies differences in expression patterns between pairs of samples, while EBSeq uses an empirical Bayesian approach to identify the most likely among multiple possible expression patterns. Using EBSeq, we considered three alternatives: (1) the null hypothesis that no samples had differential expression; (2) the alternative hypothesis that expression in the sample associated with queen development/rearing was different than the samples associated with worker development/rearing; and (3) the alternative hypothesis that expression in the sample with the queen present was different than expression in the samples with the queen removed. We used default settings except for an increased number of iterations (maxround = 40) to ensure convergence. With DESeq2, we used default settings and ran two separate analyses to identify genes with differential expression associated with queen vs. worker development and genes with differential expression associated with queen presence vs. absence. We focus on the EBSeq results for subsequent analyses because EBSeq is most suitable for our study, but we also report DESeq2 results because the DEseq2 analysis was more conservative for identifying genes associated with caste (Fig. S1), but not for genes associated with queen presence (Fig. S2). Subsequent analyses were qualitatively similar following either EBSeq or DESeq2 differential expression analysis. Finally, we annotated transcripts with Blast2go (Conesa et al. 2005) and performed Gene Ontology (GO) enrichment analysis with the GOstats R package (Falcon and Gentleman 2007). Venn diagrams of differentially expressed genes were constructed with the VennDiagram R package (Chen and Boutros 2011).

### Molecular evolution analysis

To study patterns of molecular evolution at our identified differentially expressed nurse and larval genes, we compared the estimated strength of selection on the genes since the divergence of *A. mellifera* and *A. cerana*, ~5–25 Ma (Harpur et al. 2014). Specifically, we used a new database of estimates of the population size‐scaled selection coefficient *γ* (*γ *= 2*N*_*e*_ *s*; the product of effective population size and the average selection coefficient) (Harpur et al. 2014). These estimates are based on polymorphism at synonymous and nonsynonymous sites within an African *A. mellifera* population compared to fixed differences between *A. mellifera* and *A. cerana*, and thus provide more information than measures based only on fixed differences between lineages such as dN/dS (Harpur et al. 2014). We compared *γ* estimates for differentially expressed genes to background genes, which were not differentially expressed but had expression levels summed across all samples that were greater than or equal to the minimum expression levels in the list of differentially expressed genes. Finally, we compared *γ* estimates for different categories of caste‐associated genes.

## Results

### Analysis of nurse behavioral and physiological specialization

To clarify the potential specialization of nurses on provisioning worker vs. queen cells, we observed the feeding behavior of individually marked workers in two colonies over a period of 4 days during emergency queen rearing, for a total of 40 h of observation. Nurses observed provisioning queen cells were on average 1.6 days younger than nurses observed provisioning worker cells (9.3 vs. 10.9 days, respectively; Fig. S1) (glm, quasipoisson residuals, *t *=* *2.60, *df* = 191, *P *=* *0.01). Of individual nurses observed for multiple feeding events within a single day, 37 provisioned only queen cells or worker cells and 13 provisioned both. Of those observed multiple times among days, 7 provisioned only queen cells or worker cells and 11 provisioned both. Thus, nurses tended to provision only queen cells or worker cells within days but not across days (Fisher's exact test, *P *<* *0.001). We also measured the size of nurse HPG acini as an indicator of gland activity (Ohashi et al. 2000). Using residuals after controlling for differences among replicate colonies, royal nurses had larger HPG acini than worker nurses in queen present conditions (Tukey contrast with glm, *z *=* *2.94, *P *=* *0.009), but all other comparisons were not different (Tukey contrasts with glm, all *P *>* *0.19) (Fig. S2).

### Differential expression analysis

**Figure 2:**
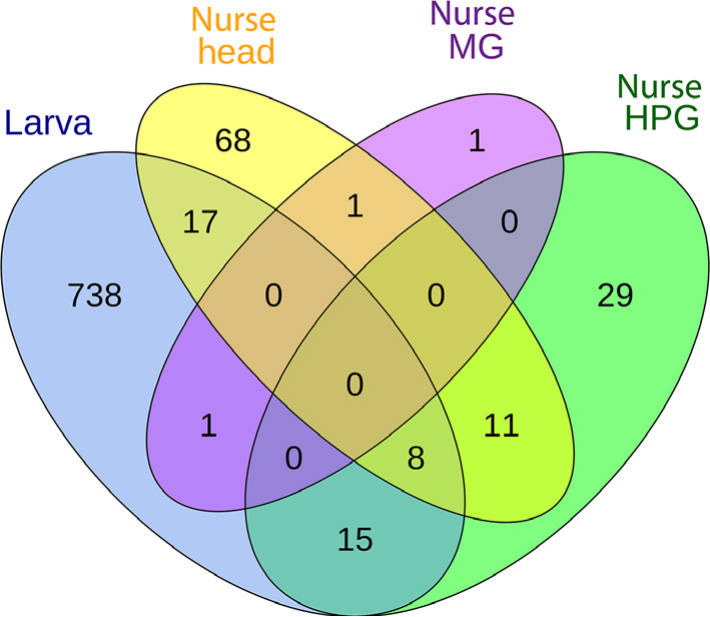
Overlap of genes with caste‐associated expression patterns that were identified from four larval and nurse tissues. Analysis was based on whole larval samples (Larva) and three separate nurse tissues: the mandibular gland (MG) and hypopharyngeal gland (HPG), which are two nurse head glands that are the main sources of brood food, and the remaining head tissue (Head), which mainly includes brain tissue. Results are based on EBSeq differential expression analysis.

We identified 950 differentially expressed genes putatively associated with whether larvae developed into new queens or workers (Table 1; Table S2). The majority of these genes (82%; 779/950) were differentially expressed in the larvae themselves, depending on whether the larvae were queen‐ or worker‐destined larvae. A total of 18% (171/950) were differentially expressed in *nurses* collected while feeding queen‐destined larvae compared to nurses collected while feeding worker‐destined larvae (3 expressed in MG, 105 H, and 63 HPG) (Table S2). Overlap of differentially expressed genes associated with caste development is shown by tissue type in Figure 2.

**Table 1:** Select highly expressed nurse genes putatively associated with larval caste development

Gene	Log10 expression	Log2 Fold Change	Tissue	Upregulated	Annotation	Function	RJ proteome
GB53576	5.80	1.36	H	Royal	Apisimin precursor	Antimicrobial	Yes
GB53576	5.80	−1.39	MG	Worker	Apisimin precursor	Antimicrobial	Yes
GB41428	4.10	1.65	HPG	Royal	Defensin‐1 preproprotein	Antimicrobial	Yes
GB51223	2.81	1.91	HPG	Royal	Hymenoptaecin preproprotein	Antimicrobial	Yes
GB51223	2.51	2.52	H	Royal	Hymenoptaecin preproprotein	Antimicrobial	Yes
GB47318	1.71	1.52	HPG	Royal	Abaecin precursor	Antimicrobial	
GB53578	3.98	1.18	H	Royal	Glucosylceramidase‐like isoform 1	Metabolic activity	Yes
GB43805	2.93	0.82	H	Royal	Membrane metallo‐endopeptidase‐like 1‐like	Metabolic activity	Yes
GB55204	5.58	0.88	H	Royal	Major royal jelly protein 3	Nutritional	Yes
GB45796	5.38	1.07	H	Royal	Major royal jelly protein 3‐ partial	Nutritional	Yes
GB50012	3.73	0.99	HPG	Royal	Hypothetical protein LOC726323	Unknown	Yes
GB50012	3.36	1.51	H	Royal	Hypothetical protein LOC726323	Unknown	Yes
GB49583	2.36	1.50	HPG	Royal	40s ribosomal protein s14	Protein synthesis	
GB50709	2.00	1.22	HPG	Royal	40s ribosomal protein s19a‐like	Protein synthesis	
GB45374	2.99	0.66	HPG	Royal	40s ribosomal protein s23‐like	Protein synthesis	
GB50356	3.42	1.58	HPG	Royal	60s acidic ribosomal protein p2‐like	Protein synthesis	
GB52789	2.61	1.80	HPG	Royal	60s ribosomal protein l22 isoform 1	Protein synthesis	

Mean expression across conditions (i.e., mean normalized counts) is shown as Log10 expression for each gene, relative expression in royal nurse tissues vs. worker nurse tissues is shown as Log2 Fold Change, tissue (H = head tissue, MG = mandibular gland tissue), whether the gene was upregulated in royal nurses or worker nurses, annotation, inferred functional category, and whether the encoded protein has been identified in the royal jelly proteome and thus assumed to be secreted from nurse glands to the brood food.

**Figure 3:**
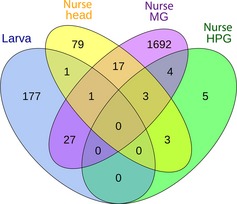
Overlap of genes with expression patterns that depended on queen presence for the four larval and nurse tissues. Results are based on EBSeq differential expression analysis.

We also identified 2069 genes that were differentially expressed depending on queen presence, that is, whether the mother queen was present or removed, irrespective of larval caste fate or nurse behavior (Table 2; Table S3). A total of 90% (1863/2069) were expressed in nurse tissues, especially MG (1744 MG, 105 H, 15 HPG), and 206 were expressed in larval tissue. Overlap of differentially expressed genes associated with queen presence is shown by tissue type in Figure 3.

**Table 2:** Select highly expressed nurse genes putatively associated with queen presence

Gene	Log10 expression	Log2 Fold Change	Tissue	Upregulated	Annotation	Function	RJ proteome
GB55205	5.42	0.85	H	Queen present	Major royal jelly protein 1 precursor	Nutrition	Yes
GB55212	4.70	1.21	H	Queen present	Major royal jelly protein 2 precursor	Nutrition	Yes
GB55211	3.94	0.84	H	Queen present	Major royal jelly protein 2 precursor	Nutrition	Yes
GB55206	4.03	0.75	H	Queen present	Major royal jelly protein 4 precursor	Nutrition	Yes
GB55208	3.99	0.79	H	Queen present	Major royal jelly protein 5	Nutrition	Yes
GB55209	5.17	0.84	H	Queen present	Major royal jelly protein 5 precursor	Nutrition	Yes
GB55207	3.21	0.86	H	Queen present	Major royal jelly protein 6 precursor	Nutrition	Yes
GB55213	4.10	0.66	H	Queen present	Major royal jelly protein 7 precursor	Nutrition	Yes
GB55215	2.14	1.44	H	Queen present	Major royal jelly protein 9 precursor	Nutrition	Yes
GB55729	2.89	−1.03	MG	Queen absent	Major royal jelly protein 1	Nutrition	Yes
GB45797	2.39	1.79	MG	Queen present	Major royal jelly protein 1‐ partial	Nutrition	Yes
GB55205	5.72	−1.39	MG	Queen absent	Major royal jelly protein 1 precursor	Nutrition	Yes
GB45796	5.39	0.77	MG	Queen present	Major royal jelly protein 3‐ partial	Nutrition	Yes
GB55208	4.25	1.93	MG	Queen present	Major royal jelly protein 5	Nutrition	Yes
GB55209	5.28	0.79	MG	Queen present	Major royal jelly protein 5 precursor	Nutrition	Yes
GB55207	3.28	−0.48	MG	Queen absent	Major royal jelly protein 6 precursor	Nutrition	Yes
GB55213	4.39	−0.25	MG	Queen absent	Major royal jelly protein 7 precursor	Nutrition	Yes

Mean expression across conditions (i.e., mean normalized counts) is shown as Log10 expression for each gene, relative expression in nurse tissues in queen absent vs. queen present conditions is shown as Log2 Fold Change, tissue (H = head tissue, MG = mandibular gland tissue), whether the gene was upregulated in queen present or queen absent colony conditions, annotation, inferred functional category, and whether the encoded protein has been identified in the royal jelly proteome, and thus assumed to be secreted from nurse glands to the brood food.

As expected, genes whose proteins make up the primary components of royal jelly, including 8 of the 9 major royal jelly proteins (MRJPs), were among the most highly expressed genes in nurse tissues (Table S1) and were also differentially expressed (Tables 1 and 2). However, of the mrjp genes, only the expression of mrjp3, which has previously been implicated as promoting queen development (Huang et al. 2012), depended on nurse behavior: It was upregulated in the head tissue of royal nurses (Table 1). All eight differentially expressed mrjp genes, including mrjp1, also implicated as promoting queen development (Kamakura 2011), were differentially expressed in nurse mandibular glands or head tissue, depending on queen presence. Most were upregulated in the queen removed condition (Table 2), presumably related to colony‐level changes associated with the rapid shift to emergency queen rearing. Notably, 4 of the 6 described honey bee antimicrobial peptides (Evans et al. 2006) (defensin 1, abaecin, hymenoptaecin, and apisimin) were upregulated in the HPG and/or the head tissues of nurses feeding queen‐destined larvae (Table 1), and caste‐associated nurse‐expressed genes were enriched for Gene Ontology terms for immune function (Table S4). Hymenoptaecin and another antimicrobial peptide, apidaecin, were also upregulated in queen‐destined larvae. Altogether, these results suggest that queen‐ and worker‐destined larvae may require different levels of antimicrobial peptides, some of which may be produced by nurse workers and transferred to larvae through royal jelly (Schonleben et al. 2007; Furusawa et al. 2008; Zhang et al. 2014).

Considering the top 25 most highly expressed genes for each tissue (Table S1), 40% (10/25) were shared among the nurse tissues. Many of these highly expressed nurse genes are known to have protein products that are present in royal jelly (Schonleben et al. 2007; Furusawa et al. 2008; Zhang et al. 2014) (Table S1). Approximately one‐third of each set of most highly expressed genes was unique to each nurse tissue, whereas ~90% (22/25) of the most highly expressed larval genes were unique to larvae (Fig. S5).

GO enrichment analysis for differentially expressed genes associated with caste or queen presence is shown by tissue type in Tables S4 and S5, respectively. Among genes associated with caste, genes differentially expressed in nurse HPG tissue were enriched for GO terms associated with translation and several categories associated with immune function; nurse head tissue genes showed a weaker signal of enrichment for a range of GO terms, including signaling; and larval‐expressed genes were enriched for terms such as metabolic processes and chromatin assembly. Among genes that were differentially expressed depending on queen presence, nurse MG genes were enriched for a range of terms including translation and transcription, macromolecular biosynthesis, signal transduction, metabolism, and immune response; nurse head tissue genes were enriched for immune system function, brain development, and chromatin assembly; and larval genes were enriched for terms such as response to oxidative stress and metabolism.

### Molecular evolution analysis

**Figure 4:**
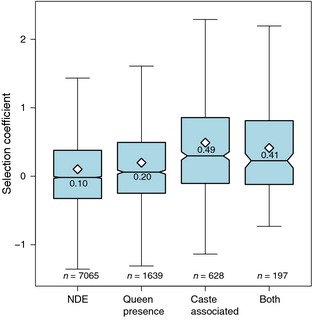
Box and whisker plots of population size‐calibrated selection coefficients (*γ*) for nondifferentially expressed genes (NDE), nurse and larval genes with expression associated with queen presence (“queen presence”), nurse and larval genes with expression associated with caste development (“caste”), and nurse and larval genes with expression associated with both queen presence and caste in different tissues (“both”). Genes that were nondifferentially expressed or had expression only dependent on queen presence had lower selection coefficients than genes with caste‐associated expression or both caste‐ and queen presence‐associated expressions. Means are indicated by white diamonds and also printed in each box. Outliers are removed for clarity.

Differentially expressed genes, whether associated with caste development or queen presence, had higher average selection coefficients (*γ*) than nondifferentially expressed genes (Fig. 4; glm on log‐transformed gamma estimates, all *P *< 10^−8^), and furthermore, genes with expression associated with caste or both caste and queen presence had higher *γ* than genes with expression only associated with queen presence (Fig. 4; Tukey contrasts, both *P *< 10^−4^).

**Figure 5:**
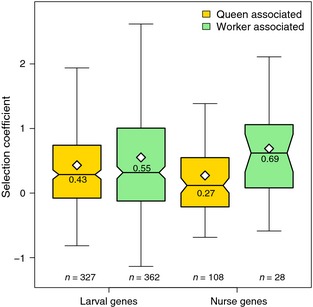
Box and whisker plots of population size‐calibrated selection coefficients (*γ*) for nurse and larval differentially expressed genes associated with caste. Genes are grouped by tissue type (larval vs. nurse tissues), and whether they were upregulated in queen larvae or royal nurses (queen associated, yellow boxes) or they were upregulated in worker larvae or worker nurses (worker associated, green boxes). On average, larval and nurse genes with worker‐associated expression had higher estimated selection coefficients than genes with queen‐associated expression. Nurse‐ and larval‐expressed genes did not have different mean selection coefficients. Means are indicated by white diamonds and also printed in each box. Outliers are removed for clarity.

Next, we focused on genes with caste‐associated expression. To further compare patterns of molecular evolution at genes associated with queen vs. worker production, we defined genes upregulated in queen larvae or royal nurse tissues as “queen‐associated genes” and genes upregulated in worker larvae or worker nurse tissues as “worker‐associated genes.” Mean *γ* for worker‐associated genes was higher than queen‐associated genes (glm with log‐link on *γ + 2* values, *t *=* *2.47, *df *= 824*, P *=* *0.014), consistent with our second prediction that all genes associated with worker development should experience more rapid molecular evolution. However, *γ* was not different for caste‐associated genes that were expressed in larval versus nurse tissues (*P *=* *0.33) (Fig. 5), inconsistent with our first prediction that nurse‐expressed genes associated with caste should be shaped more by indirect selection and thus experience more rapid molecular evolution. When only considering nurse‐expressed genes, *γ* was higher for queen‐associated vs. worker‐associated genes (*t *=* *3.71, df = 135, *P *=* *0.0076), but *γ* was not significantly different when only considering larval‐expressed genes (*t *=* *1.78, df = 688, *P *=* *0.076).

## Discussion

We simultaneously studied the gene expression profiles of two classes of socially interacting individuals – developing larvae and their caregiving adult nurses – in order to identify genes expressed in larvae and their nurses that are associated with larval caste development. This approach is based on the interacting phenotype conceptual framework, whereby an individual's traits depend directly on its own genes (direct genetic effects) and indirectly on its social partners' genes (indirect genetic effects) (Moore et al. 1997; Bleakley et al. 2010; McGlothlin et al. 2010; Wolf and Moore 2010). While this framework is regularly used in quantitative genetic studies of the contribution of heritable indirect effects to trait *variation*, as far as we know, this study is the first to use a transcriptomic extension of this framework to identify genes with putative direct and indirect effects on trait *expression* (previous transcriptomic studies considering indirect genetic effects have treated gene expression profiles as variable traits that are influenced by genetic variation for direct and indirect effects, e.g., Wang et al. 2008; Gempe et al. 2012). Thus, our approach seeks to uncover the full network of genes underlying social trait expression and was proposed as a means to study the molecular basis of social interactions (Linksvayer et al. 2012). Our approach is analogous to recent studies of the molecular basis of host–parasite interactions that also use RNA sequencing to simultaneously study the gene expression profiles of interacting organisms (Tiemey et al. 2012; Westermann et al. 2012).

We identified hundreds of genes that were differentially expressed in both developing honey bee larvae and caregiving nurse workers that were associated with whether the larvae were destined to develop as new queens or workers. The majority of these genes (82%; 779/950) were differentially expressed in the larvae themselves, depending on larval caste trajectory. These larval‐expressed genes are putatively directly involved in the expression of developmental plasticity underlying queen–worker dimorphism, as identified by previous studies of the endogenous molecular basis of queen–worker development (Evans and Wheeler 1999; Barchuk et al. 2007; Foret et al. 2012). A total of 18% (171/950) of genes with expression patterns associated with queen versus worker production were differentially expressed in nurse tissues, depending on whether the nurses were royal nurses or worker nurses. These differentially expressed nurse genes associated with caste development provide putative examples of genes with indirect genetic effects, which occur when genes expressed in one individual affect traits expressed by a social partner (Wolf and Moore 2010). Many of the highly expressed and caste‐associated genes we identified have protein products that have previously been found in royal jelly (Schonleben et al. 2007; Furusawa et al. 2008; Zhang et al. 2014). These nurse‐produced royal jelly components are directly fed to developing larvae, providing a direct mode of action of social regulation of larval caste fate (Kamakura 2011; Huang et al. 2012). Other caste‐associated nurse genes with protein products that are not known to be secreted into royal jelly may have a more circuitous effect on larval caste fate through their effect on nurse worker physiology or provisioning behavior (Haydak 1970; Brouwers et al. 1987; Hatch et al. 1999).

In accordance with previous social insect transcriptomic studies (Grozinger et al. 2003; Malka et al. 2014; Manfredini et al. 2014), we also identified many nurse‐expressed genes with expression patterns dependent on queen presence. At the colony level, queen removal or death results in a rapid shift from exclusively worker rearing to emergency rearing of a handful of new queens. Thus, these nurse genes that initially respond to queen loss may be associated with the production of new queens and may represent additional caste‐associated nurse genes. Over longer periods of time following queen loss and unsuccessful queen rearing, additional sets of genes change expression patterns in a subset of workers that activate their ovaries and begin laying unfertilized drone eggs (Thompson et al. 2008; Cardoen et al. 2011).

We predicted that nurse genes with putative indirect effects on larval caste fate would experience relaxed selective constraint and have higher estimated selection coefficients than larval genes with putative direct effects on larval caste fate (Linksvayer and Wade 2009). However, we observed no mean difference between larval‐ and nurse‐expressed caste‐associated genes. Secondly, we predicted that at a finer scale, both larval and nurse genes associated with worker development would experience relaxed selective constraint and higher selection coefficients relative to larval and nurse genes associated with queen development. As workers honey bees are facultatively sterile, worker‐associated genes should ultimately be shaped primarily by indirect selection (i.e., kin selection). This prediction was supported: Among putatively caste‐associated genes, genes upregulated in worker larvae and worker nurses had higher selection coefficients than genes upregulated in queen larvae and royal nurses (Fig. 5). We also found when considering both larval‐ and nurse‐expressed genes together, genes with putative caste‐associated expression had higher estimated selection coefficients than nondifferentially expressed genes and genes with expression dependent on queen presence (Fig. 4). Altogether, our results suggest that both genes with putative direct and indirect effects on larval development – especially those associated with worker development – have experienced elevated rates of molecular evolution and have contributed to the evolution of the honey bee caste system. These results are consistent with two recent honey bee studies showing that genes associated with *adult* worker traits are also rapidly evolving. The first study shows that genes encoding proteins that are more highly expressed in adult honey bee workers compared to adult queens have experience stronger selection (Harpur et al. 2014). The second study finds that the most highly expressed genes in specialized adult tissues with derived social functions, such as the hypopharyngeal and mandibular glands, tend to be very rapidly evolving, taxonomically restricted genes (Jasper et al. 2014).

We found some evidence for short‐term behavioral and physiological specialization of nurses on feeding queens versus workers, besides the broad gene expression differences we observed. On average, royal nurses also had larger hypopharyngeal glands and were 1.5 days younger than worker nurses (Figs. S1 and S2). Previous studies have shown that nurse gland size and activity (Ohashi et al. 2000), as well as the composition of nurse glandular secretions (Haydak 1970), and patterns of nurse brain gene expression (Whitfield et al. 2006) all vary with nurse age and social environment. While it is not clear how exactly these differences are related to the observed differences in nurse provisioning behavior, individually marked nurses did tend to specialize on feeding either queen or worker cells within a day, but not across multiple days. Longer‐term tracking of individuals during queen rearing will be necessary to definitively demonstrate the degree to which nurse specialization occurs. The key point for this study of colony‐level caste regulation is that queen‐ vs. worker‐destined larvae interact with nurses that are on average transcriptionally and physiologically distinct, resulting in distinct rearing environments and alternate caste developmental trajectories.

## Conclusions

Quantitative genetic studies using the interacting phenotype framework in a range of organisms, from plants to social insects to mammals, have shown that indirect genetic effects make strong contributions to heritable variation and can strongly affect evolutionary dynamics (Bleakley et al. 2010; Wolf and Moore 2010). Our study demonstrates that the interacting phenotype framework is readily extended to consider the full transcriptional architecture and molecular basis of complex social traits, including genes with both direct and indirect effects, that is, the “social interactome” – as opposed to only focusing on the subset of these genes that currently harbor segregating variation and contribute to observed patterns of phenotypic variation. Our results hint at a much broader contribution of nurse‐expressed genes to the colony‐level gene network regulating caste development than has previously been considered, consistent with the notion that caste is influenced by multiple nurse‐produced and nurse‐regulated factors (Linksvayer et al. 2011; Leimar et al. 2012; Buttstedt et al. 2014).

Increasingly, studies have shown how the gene expression profiles of many animals, including honey bees, ants, fruit flies, and cichlid fish strongly depend on the social environment (Grozinger et al. 2003; Robinson et al. 2008; Malka et al. 2014; Manfredini et al. 2014). Social environments in turn depend on the traits – and genes – of social partners (Wolf and Moore 2010). With such interdependence, studies such as ours which simultaneously study of the traits and genes of interacting partners are likely needed to capture the full dynamic social interplay affecting behavior, physiology, development, trait expression, and fitness (Johnson and Linksvayer 2010; Linksvayer et al. 2012).

## Data Accessibility

Raw RNA seq reads are available in the NCBI Sequence Read Archive, BioProject ID: PRJNA295415.

Read counts per gene per sample, as well as summaries of total mapped reads per sample are available at Dryad: doi:10.5061/dryad.c57h7.

Raw behavioral scan data are available at Dryad: doi:10.5061/dryad.c57h7.

## Conflict of Interest

None declared.

## Supporting information


**Figure S1.** Box and whisker plot of the age of individually marked “royal nurses” that were observed feeding queen‐destined larvae in queen cells compared to “worker nurses” that were observed feeding worker‐destined larvae in worker cells. Outliers are removed for clarity.

**Figure S2.** Box and whisker plot of residual nurse hypopharyngeal gland acini size (*μ*m) depending on queen presence and nurse provisioning behavior. Royal nurses had larger HPG acini than worker nurses collected from colonies with a queen. Outliers are removed for clarity.

**Figure S3.** Venn diagram showing overlap of differentially expressed genes associated with caste identified by EBSeq and DESEq2. For this comparison, DESeq2 is more conservative, identifying mainly a subset of EBSeq‐identified genes.

**Figure S4.** Venn diagram showing overlap of differentially expressed genes associated with queen presence identified by EBSeq and DESEq2. For this comparison, EBSeq is somewhat more conservative than DESeq2, with less overlap than for caste‐associated expression.

**Figure S5.** Venn diagram showing overlap of the top 25 most highly expressed genes for each tissue.

**Table S1.** The top 25 most highly expressed genes by tissue (HPG = nurse hypopharyngeal gland tissue; H = remaining nurse head tissue; L = larval tissue; MG = nurse mandibular gland tissue). Mean expression level (i.e., mean normalized counts) is shown as Log10 expression. Genes whose proteins have been identified in studies of the royal jelly proteome are identified.

**Table S2.** All differentially expressed genes putatively associated with caste development, identified by EBSeq or DESeq2, grouped by tissue and sorted by expression level. Mean expression across conditions (i.e., mean normalized counts) is shown; log2 Fold Change indicates the log2 fold change when comparing queen‐associated gene expression to worker‐associated gene expression; lfcSE shows the standard error for log2 Fold Change; the columns DESeq2 and EBseq indicate whether the genes were identified as being differentially expressed with DESeq2 and EBseq analysis, respectively; the column “NL” indicates whether the gene was differentially expressed in nurse (N) or larval (L) tissue; “QW” indicates whether the gene was upregulated in worker larvae or worker nurse tissues (W) or queen larvae or royal nurses (Q).

**Table S3.** All differentially expressed genes putatively associated with queen presence, identified by EBSeq or DESeq2, grouped by tissue and sorted by expression level, as in Table S2.

**Table S4.** GO analysis for caste‐associated genes by tissue.

**Table S5.** GO analysis for queen‐presence associated genes by tissue.
