# Supplementary figures and images for: The transcriptomic and evolutionary signature of social interactions regulating honey bee caste development
Source: Ecol Evol. 2015 Oct 8;5(21):4795–807. doi: 10.1002/ece3.1720 (PMC4662310; doi:10.1002/ece3.1720)

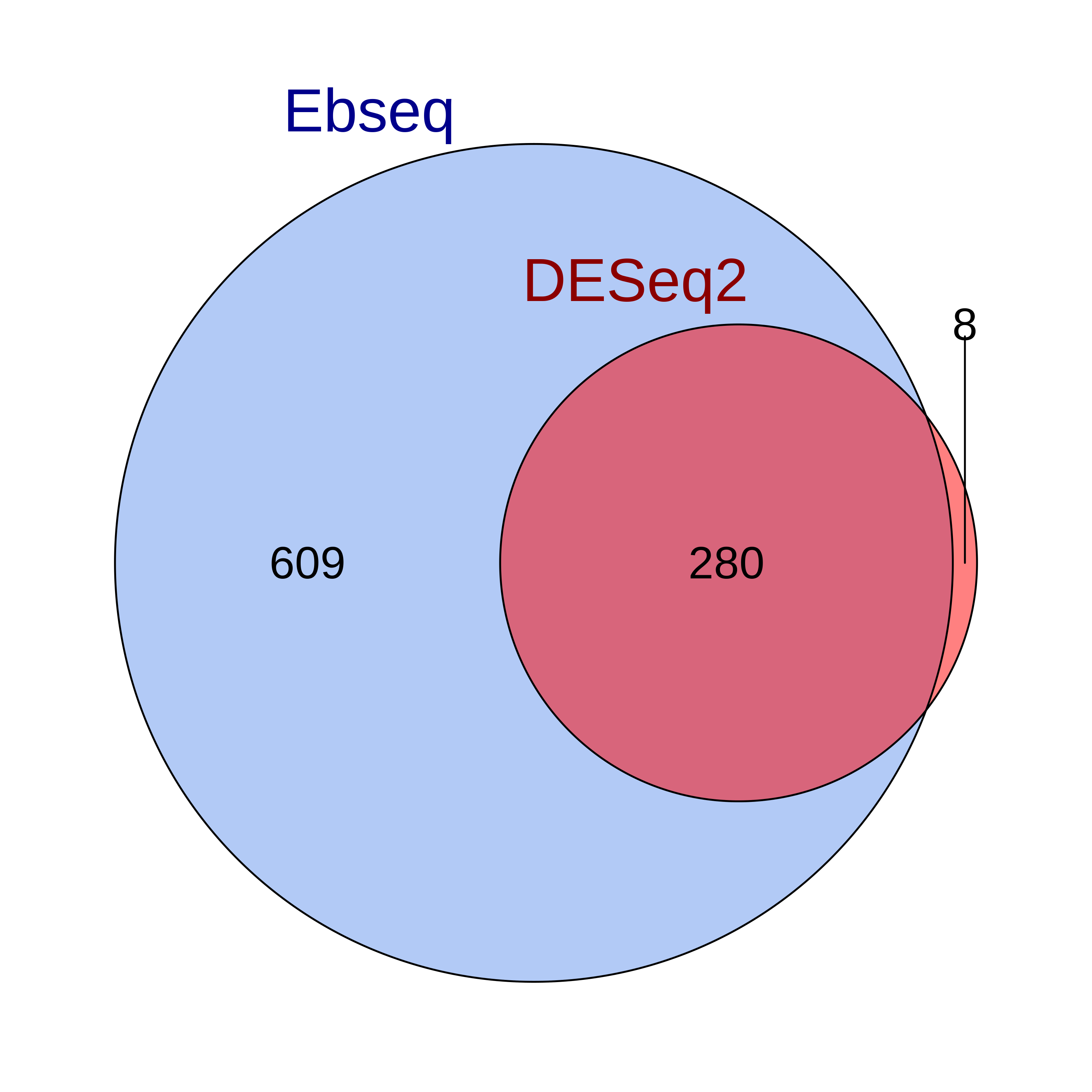

Supplement: Supplementary file 3 — Figure S3. Venn diagram showing overlap of differentially expressed genes associated with caste identified by EBSeq and DESEq2. For this comparison, DESeq2 is more conservative, identifying mainly a subset of EBSeq‐identified genes. [file ECE3-5-4795-s003.tiff]

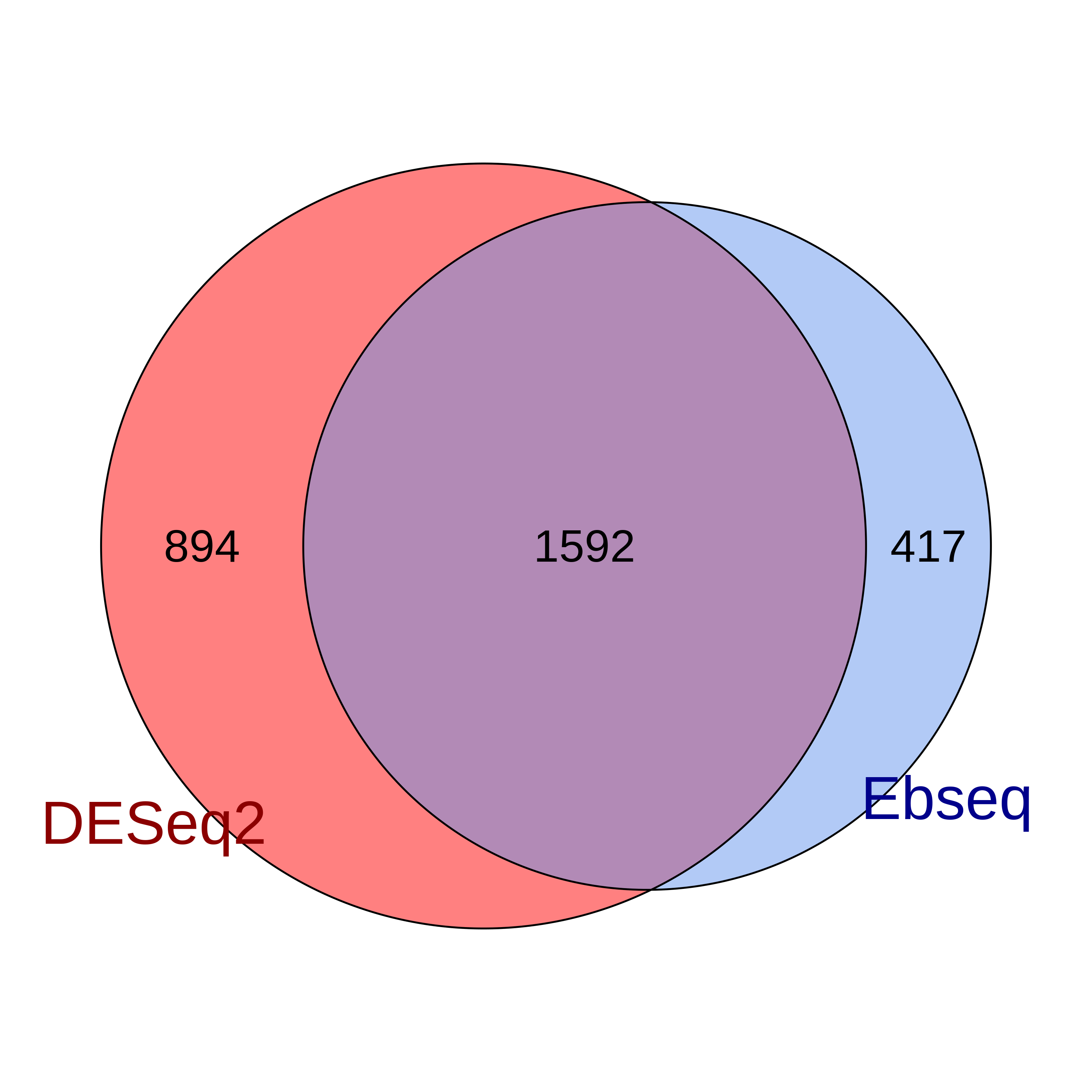

Supplement: Supplementary file 4 — Figure S4. Venn diagram showing overlap of differentially expressed genes associated with queen presence identified by EBSeq and DESEq2. For this comparison, EBSeq is somewhat more conservative than DESeq2, with less overlap than for caste‐associated expression. [file ECE3-5-4795-s004.tiff]

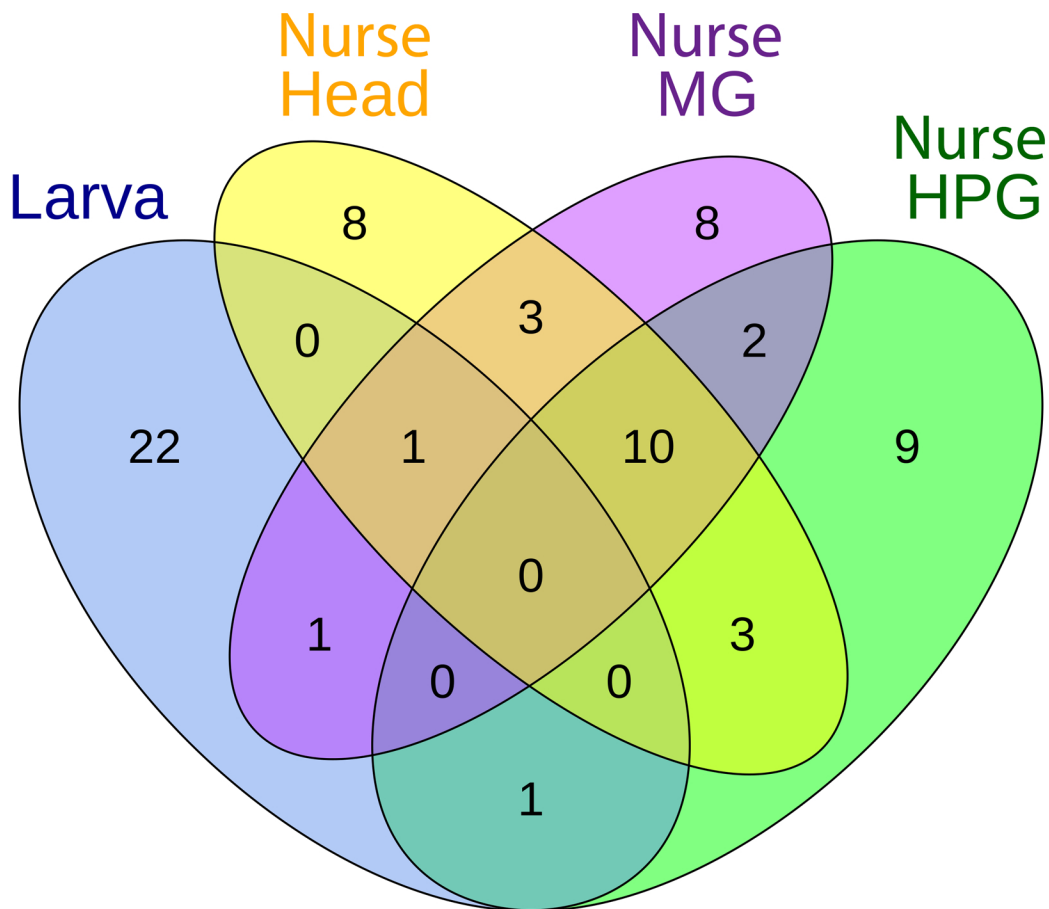

Supplement: Supplementary file 5 — Figure S5. Venn diagram showing overlap of the top 25 most highly expressed genes for each tissue. [file ECE3-5-4795-s005.pdf]
